# Development of Nutrient Rich Morning Meals for Students by Concept of Tapas

**DOI:** 10.3390/foods13213432

**Published:** 2024-10-28

**Authors:** Valentina Obradović, Maja Ergović Ravančić, Helena Marčetić, Nikola Vuksanović, Svjetlana Škrabal

**Affiliations:** 1 Faculty of Tourism and Rural Development in Požega, Josip Juraj Strossmayer University of Osijek, Vukovarska 17, 34 000 Požega, Croatia; mergovic@ftrr.hr (M.E.R.); hmarcetic@ftrr.hr (H.M.); sskrabal@ftrr.hr (S.Š.); 2 Faculty of Management, University “Union-Nikola Tesla”, 21205 Sremski Karlovci, Serbia; nikola.vuksanovic@famns.edu.rs

**Keywords:** tapas, student, nutrition

## Abstract

A correlation between nutritional status and academic achievement has been established by many studies, but students′ eating habits often do not meet nutritional recommendations. Breakfast consumption has a positive effect on cognitively demanding tasks and results in better attention and memory. The goal of this work was to develop well-balanced, nutritionally rich morning meals for students based on the concept of tapas, a combination of several different small meals composed of different ingredients. An introductory survey about students’ morning eating habits was conducted among students of the Faculty of Tourism and Rural Development in Požega, Croatia. Forty-six full-time students (9.50% of total number of students at the faculty) participated in the survey. Sixteen types of tapas were prepared combining ingredients which students rated as desirable (cheese, prosciutto, peppers, milk spread) and undesirable (blue fish, dry fruit, cauliflower, chickpeas) in the initial survey. Tapas (one tapa = one sample) were scored by a sensory panel of 16 students, and nutritional value of the samples was assessed by web application Program Prehrane^®^ (The Nutrition Program). All samples except two were scored as desirable by more than 60% of students, meaning that even ingredients which have been initially scored as undesirable, can be incorporated into desirable meal when properly combined. Based on the analysis of energy and nutritional value of samples, students should combine 3 to 5 different tapas to fulfill their energy needs for breakfast or 1 to 2 different tapas for a morning snack. Developed tapas can provide a perfectly balanced meal rich in different micronutrients because they include ingredients which students normally do not include in their breakfast. Especially important ingredients were blue fish rich in unsaturated acids and selenium, nuts rich in selenium and vitamin E, and red peppers rich in vitamin C and carotenoids. Tapas containing cheese and prosciutto, students’ favorite ingredients, had the highest level of sodium and fats, but not above recommended values when combined with other tapas.

## 1. Introduction

Adolescence is a transition between childhood and adulthood and university life is an important period during that life change. Moving away from home, exposure to new influences and making their own decisions change many aspects of students′ lives [1]. Unlike living in a family environment, they need to decide what to eat and where to eat [2]. Stress, short sleep, economic limitations and lack of time are some of the factors influencing their new eating habits [3,4]. Unhealthy eating patterns such as eating less fruits and vegetables, but more convenience food and skipping meals, especially breakfast, leads to inadequate nutrient intake [5,6,7,8]. A statistically significant correlation between eating habits/nutritional status and academic achievement has been proven [6,7,8]. Additionally, improper diet together with physical activity can be considered as risk factors for different chronic diseases in later life. Universities should promote a healthy lifestyle not only as a short-term benefit during academic life, but also as for disease prevention [9,10]. There is a positive correlation between nutrition knowledge and proper food habits [11,12,13]. Interventions in terms of different education have been proven to be successful in improvement of students′ dietary habits [14]. Refs. [15,16] concluded that students are ideal targets for lifestyle interventions. They are in a learning environment and still at an age where health behaviours can be improved. To promote the habit of eating healthy meals, availability of such food is the first step. Most universities have dining facilities, and they should offer nutritious and varied menus [10,17].

According to the survey about dietary habits of Croatian students published by [18], Croatian students are no exception to the above-mentioned findings from other countries. There were only 26% of respondents whose diet adhered to dietary guidelines, although participants had better knowledge than the general population due to the field of their studies.

Correlation between nutrients and cognitive ability has been widely studied in past decades. It has been clearly determined that nutritional status directly affects brain and neuronal functioning such as cognitive processes like memory and learning ability, emotions, behaviours, and neuronal plasticity. Different micro and macronutrients are directly related to the mentioned functions [19,20,21]. Although breakfast is often described as “the most important meal of the day”, the relationship between breakfast consumption/composition and the cognitive performance of children and adolescents still requires additional research. There is a significant inconsistency and heterogenicity in research designs and methodologies among the studies [22,23,24]. However, most of the studies highlight the advantage of breakfast consumption for memory [25], primarily delayed recall [24], and for better results in attention [25] and cognitively demanding tasks [26].

In that light, the goal of this paper was to develop meals that would be available at campus restaurant (Josip Juraj Strossmayer University, Faculty of Tourism and Rural Development in Požega, Croatia), which would be served during morning hours for breakfast or morning snack, and to obtain feedback from the students about the offered meals. The morning meals have been chosen in this survey for the following reasons: (1) most of the students eat at the campus restaurant in the morning hours because the daily schedule of full-time students normally starts at 8 a.m.; (2) beneficial short-term effects of breakfast due to energy supply after overnight fasting period which reduces hunger [23,27]; and (3) potential long-term benefits in improvement of diet quality and nutrient balance [23]. The meals have the concept of tapas, small simple dishes, so each student could combine several of them to fulfil the caloric needs and to expand the nutritional value of the meal. This concept allows for a variety of combinations and personalization of a meal, as well as application to other contexts and regions. The intention was to develop simple recipes so the staff in the restaurant would not have to invest too much time in preparation. At the end of the research, the plan is to implement the best scored meals as everyday offerings at the campus restaurant.

## 2. Materials and Methods

### 2.1. Starting Survey

The initial questionnaire consisted of questions related to the respondent′s gender and field of study, followed by questions related to the respondent′s eating habits and preferences for certain foods. The research was anonymous and voluntary, and conducted using Microsoft Forms (Microsoft Office 365, Microsoft Corporation, Redmond, WA, USA). The chosen method for testing the respondents′ preference for certain foods was the categorical hedonic scale of ten degrees (1 extremely unacceptable, 10 extremely acceptable), used to determine the overall acceptability of individual ingredients. The goal was to include ingredients that were rated as highly desirable in the students’ diet, as well as those that were scored as undesirable. The 46 undergraduate students (9 male and 37 female students) at the Faculty of Tourism and Rural Development in Požega, from different fields of study (31 enogastronomy study, 15 social sciences field of the study), participated in the initial examination. These 46 students represent 9.50% of a total number of students at the Faculty of Tourism and Rural development in Požega. However, 57% of all students are part-time (working) students who do not eat at the campus restaurant because they attend classes in the evening and on weekends. Considering only the full-time students at the faculty who eat at the campus restaurant, the sample size of 46 students represents 22% of the student population, which is sufficient for generalizability. Out of all students, there are 28% of male students and 72% of female students. The Faculty of Tourism and Rural Development is located about 100 km from the home university in the heart of a rural area that is struggling with depopulation; the small number of students at the faculty is a consequence of the wider region in which the faculty is located.

### 2.2. Preparation of Meals

After the trial preparation of various dishes, 16 types of tapas were determined for further research. The key idea was to include ingredients that students do not normally consume for breakfast and do not consider desirable, but have high nutritional value, such as blue fish, vegetables, nuts, and dried fruits. All ingredients were bought at the local supermarket. Tapas were prepared in the kitchen practicum at the Faculty of Tourism and Rural Development according to Table S1 provided with the Supplementary Material files.

### 2.3. Sensory Analysis of Meals

To describe the sensory profile of individual tapas (one tapa = one sample), the panel consisted of 16 sensory evaluators of different genders and study. All members of the sensory panel have previously participated in the initial examination. Prior to the evaluation, training and group discussions were conducted in one session of 30 min with a reference product, during which the list of properties and the intensities of individual properties were defined. Appearance was described based on the properties of color, visual consistency and surface. The intensity of the smell was also evaluated. Taste was described based on the intensity of bitterness, fullness in the mouth, sandiness, consistency, salinity, acidity, astringency, spiciness, sweetness and aftertaste. Texture was described based on the viscosity and film in the mouth. The overall acceptability of the samples was scored using a nine-point categorical hedonic scale (1—Dislike extremely to 9—Like extremely) [28]. The assessment was conducted twice daily on two consecutive days in the same time period. The samples were served at room temperature. The samples were evaluated monadically, that is, one by one in a random order. Bread and tap water were used to neutralize the palate between samples. Bread served as a neutral base which absorbed any residual flavor from previous course and water was an additional palate cleanser. The results are presented as mean scores of sensory attributes.

### 2.4. Nutritional Evaluation of Meals

The Web application Program Prehrane^®^ (The Nutrition Program) (IG Prog, Rijeka, Croatia) was used for assessment of the energy value, macronutrients, and micronutrients in individual tapas (one tapa = one sample) and for comparison to dietary guidelines.

### 2.5. Data Analysis

Collected categorical data were presented by absolute frequencies, while numerical data were described by arithmetic mean and standard deviation. In the case of eating habits of respondents, the Chi-square test was used to compare categorical data between groups (between female and male respondents and between different study groups). The T-test was applied to compare the mean value of acceptability of selected foods between independent groups (for gender and study field). The Chi-square test and T-test were performed using Microsoft Excel 2019 (Microsoft Corporation, Redmond, WA, USA). To determine whether there are any statistically significant differences between the acceptability of different samples, one-way ANOVA at 95% level, by Microsoft Excel 2019 (Microsoft Corporation, Redmond, WA, USA) was used. Results were expressed as the mean value of the percentage of respondents for each grade.

## 3. Results and Discussion

### 3.1. Starting Survey

The 46 students participated in the initial research (37 females and 9 males; 31 enogastronomy students and 15 students studying social sciences). This sample represents 9.50% of the total student population at the faculty, but 22% of full-time students who eat at the campus restaurant. Table 1 presents students’ eating habits during morning hours. As it can be seen, most of the students (*n* = 30) have not been familiar with the tapas concept prior to this survey. The gender of the respondents did not have significant influence (*p* = 0.9274), while the field of study significantly influenced the results of this question (*p* = 0.0498). This was expected because enogastronomy students study international cuisine during the second year, so older students are familiar with Spanish tapas. Thirty-six of 46 participants eat breakfast, and 33 out of 46 participants eat a morning snack. There were no significant differences according to gender category (*p* = 0.8620), while enogastronomy students eat breakfast significantly more often than social science students (*p* = 0.0433). This finding is in agreement with other studies which examined the eating habits of students, in which knowledge about the importance of proper nutrition contributes to healthier eating habits [1,4,16]. The majority of students would like to have tapas for breakfast (*n* = 38) or as a morning snack (*n* = 41), regardless of gender or field of study.

**Figure 1:**
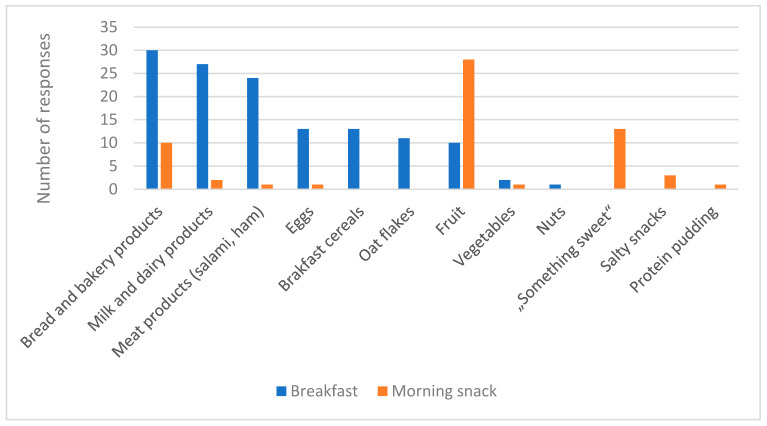
Commonly consumed foods for breakfast and morning snack (*n* = 46).

Figure 1 presents commonly consumed food by students during morning hours. Most of the students (*n* = 30) eat bread and/or other bakery products for breakfast. It is often combined with meat products like salami, ham or some dairy products like cheese spread or cheese. This type of breakfast is quite popular in Croatia and all surrounding countries. Milk and yogurt are mostly combined with breakfast cereals or oat flakes. Only 10 respondents answered that they eat fruit for breakfast. Only two respondents answered that they eat vegetables and only one student answered nuts. On the other hand, students often take fruit as a morning snack (*n* = 28), followed by “something sweet” like chocolate bars and bakery products. Students choose more convenient food, something to be prepared quickly or purchased from the bakery or store.

Students were asked to rate the acceptability of certain foods as an ingredient in tapas with grades 1–10 (1 extremely unacceptable, 10 extremely acceptable) (Table 2). Statistically significant answers between female and male respondents were only in case of leek (*p* = 0.0083), chickpeas (*p* = 0.0196) and beans (*p* = 0.0336) where male respondents gave significantly higher grades (8.56, 8.44 and 8.44, respectively) than female respondents (5.24, 5.59 and 5.78, respectively), for all three ingredients. Study field did not have a significant influence on preferences except in the case of champignons, where enogastronomy students preferred them more than social studies students (*p* = 0.0219). Looking at the overall grades, the highest rated ingredients were cheese, wheat tortilla, and whole grain tortilla, followed by whole grain bread, prosciutto, bacon, milk spread, eggs and poultry (all scored above 8 out of 10). This finding is in accordance with the previous questions regarding the foods they normally eat in the morning. Cucumber, tomato, peppers and nuts were also rated with scores above 7, although students do not include them in the breakfast very often. The least preferred ingredients were raisins, sardines, anchovies, and eggplant (all scored between 4 and 5). These answers were the starting point for the preparation of tapas recipes for several reasons. First, the intention was to prepare small snacks that were simple and quick to prepare and consume (like spreads on toast and tortillas), to maintain the daily routine of staff and students. Big changes in a short time might lead to resistance. Second, it was important to include highly-rated ingredients, which would be a lure to try a new form of a meal. Finally, nutritionally-rich ingredients that were not rated well (like sardines or dried fruit) have been included in a form that combines them with preferable foods (sardines with cream cheese, dry dates with hazelnuts and cocoa, chickpeas with cocoa and peanut butter).

### 3.2. Sensory Analysis

The results of evaluating the intensity of the appearance, smell, taste, and texture of the samples are shown in Table 3. The color of all samples was rated as acceptable by over 80% of respondents. Smell was described as pronounced by over 90% of respondents for sample number 5 (due to cabbage) and sample number 7 (due to cocoa, peanut butter and vanilla), followed by sample number 4 (due to red peppers), 6 (due to sardines), 11 (due to sardines and red peppers) and 15 (due to cinnamon), where over 70% of the respondents gave the same answer, respectively. The same samples also had pronounced mouthfeel. The bitterness of all samples was predominantly described as weak or not present except in the case of sample number 9, where 50% of students described it as pronounced due to strong chicken taste. Sandiness was pronounced for sample number 1 due to flax and sesame seeds. Mouth consistency was predominantly described as hard by 65.63% of respondents for sample number 8 (cheese bruschetta), while for all other samples it was described as soft or middle consistency. Saltiness was described as pronounced by over 60% of respondents for samples 10 (due to prosciutto) and 12 (due to parmesan and pancetta), while acidity was pronounced only in sample number 5 (due to freshly squeezed lemon juice). Astringency was mostly described as not present, but in samples number 4, 5 and 7, between 40 and 50% of respondents described it as present. The reason for that was cocoa powder in sample 7, red cabbage in sample 5 and chili powder in sample 4, whereby it was difficult for students to clearly distinguish astringency from spiciness. However, characteristic chilly hot taste was recognized in sample number 4, but, interestingly, sample number 5 was also described as hot by 75% of respondents, probably due to specific taste of cabbage. Both samples were also described as pronounced spicy by 90.63 and 87.50% of respondents. Sweetness was described as pronounced in samples 4 (due to sweet potato), 7 and 16 (due to agave syrup). All samples except 15 (apple spread) were predominantly described as dense.

Results of the nine-point categorical hedonic scale are presented in Table 4. The top four grades from 9 to 6 were grades for desirability (like extremely, like very much, like moderately and like slightly), grade 5 was neutral while the lowest four grades were for undesirability where 1 was dislike extremely. All samples were rated with the top 3 ratings by over 50% of the respondents, except the sample 5 (sample with purple cabbage), which was rated with the highest 3 ratings by 46.9% of the respondents. Samples with the highest ratings were number 8 (cheese bruschetta), 10 (tortilla with prosciutto), 12 (tortilla with Caesar souse) and 16 (pancake with cocoa and hazelnut spread) which was expected, considering students’ usual food preferences. Interestingly, the plain pancake (number 14) had low desirability grades, but when cocoa spread was added, it became highly desirable. However, the bases of cocoa and hazelnut spread were both dates. Although they were not rated in the initial survey, raisins (as other dry fruit) were rated as undesirable. Similarly, sardines were rated very low in the initial survey but when combined with other ingredients including cream cheese (sample number 6) and peppers (sample number 11) the final meal was scored as desirable product. Based on the scores, it can be concluded that even initially undesirable ingredients can be incorporated into desirable meal when properly combined. Changing dietary habits from the ground up is not a realistic option, especially for young people like students, but by carefully listening to their habits, wishes and needs, it is possible to get added nutritional value in their meals.

### 3.3. Nutritional Evaluation of Meals

All dietary recommendations have been taken from [29,30]. Daily energy intake for female students should be around 2197 kcal, while for male students it should be around 2924 kcal; the needs can vary depending on their size and physical activity. Breakfast should satisfy 20–25% of energy needs, which should be 585–731 kcal for male students and 439–549 kcal for female students, while a morning snack should satisfy 5–10% of daily energy needs, 146–292 kcal for male and 110–220 kcal for female students. As already mentioned, the idea of this paper was to develop different small, tapa-style meals, so students could combine several of them to fulfil energy needs and broaden nutrient intake. The brain is dependent on a constant supply of energy that primarily comes from glucose. Since the reserves of carbohydrates in the human body are limited, the brain’s needs depend on food intake. However, it is crucial to consider not only the amount of carbohydrates/glucose, but also the speed of the glucose entering the blood, which is called the glycaemic index. The goal is to have a slow, but constant absorption of the necessary glucose in the blood, which can be achieved by consuming food rich in fiber and by combining carbohydrates with other nutrients such as fats and proteins. It should be emphasized that proteins and fats also have an impact on cognitive function independent of blood glucose [31]. As presented in Table 5, the energy value of samples (individual tapas) varied between 41 kcal (number 14—one plain pancake) and 222 kcal (number 12—tortilla with Caesar souse). Most of the samples had an energy value between 100 and 150 kcal per serving. Depending on individual choice, students should select 3 to 5 different tapas for breakfast and 1 or 2 different tapas for morning snack. Approximately 45 to 65% of energy needs should come from carbohydrates, 10 to 20% from protein and 20 to 35% from fat.

The distribution of macronutrients in samples is shown in Table 5. Sample number 4 had the largest excess of energy originating from carbohydrates (71.8%), while at the same time it had the lowest energy value originating from fats (12.9%). Such distribution is a result of sweet potato rich in starch and sugar. The proportion of fat could be increased by the addition of olive oil; however, by combining it with other types of tapas that had an inverted distribution of macronutrients, an ideal distribution of macronutrients in the meal can be achieved.

Four samples (number 2, 6, 9 and 12) had a slightly lower ratio of energy that comes from carbohydrates than recommended (42, 39.8, 35.2 and 36.2, respectively), while in sample number 8, only 23.5% of energy came from carbohydrates. The same sample had the highest energy value from fats (53.8 %) due to different cheeses in the recipe. However, it was one of the best scored samples by sensory analysis, and it is an excellent source of calcium, zinc, retinol and vitamin C (Table 6). We recommend keeping it as a daily offering and combining it with samples number 4 and/or 7, which were low in fats and high in carbohydrates. The following samples had energy intake based on fats above recommended value: samples 2, 5, 6, 9, 12, 13, 15 and 16 (41.5, 38.6, 45.2, 45.1, 36.4, 37.2, 37.7 and 37.7, respectively). However, samples 2, 5, 6, 8 and 9 were a great source of monounsaturated fats, between 2 and 3 g. By combining several of them, it is possible to satisfy 20–25% of the recommended daily allowance for monounsaturated fats and, at the same time, not exceed the recommended upper level of cholesterol intake.

Polyunsaturated fatty acids, primarily omega-3 fatty acids are the most known chemical compounds from the group of fats in terms of their influence on cognitive functioning. Cerebral fats play no part in storing or producing energy; they participate mainly in the architecture of the cell membranes. All cells and organelles in the brain are very rich in polyunsaturated omega−3 fatty acids [31]. The most abundant omega−3 and omega−6 polyunsaturated fatty acids in the brain are docosahexaenoic acid (DHA; 22:6) and araquidonic acid (AA; 20:4), mostly obtained from diet or synthesized in the liver from dietary alpha-linolenic acid (ALA, 18:3) and linoleic acid (LA, 18:2) [20]. Polyunsaturated fatty acids, especially omega−3 fatty acids, function mainly by altering membrane lipid composition, cellular metabolism, signal transduction and even regulation of gene expression. Different compounds can influence various cellular responses and affect gene expression. Altered lipid composition of nuclear membranes may affect permeability properties, which consequently allows easier crossing of the cellular membrane for such compounds [31]. Steroid hormones are of particular interest in that context. Steroids travel through the blood using lipid carriers. Once in a membrane receptor, the steroid will detach from the carrier and translocate through the cell membrane [32]. Steroid hormones like estrogens, progesterone, androgens and glucocorticoids regulate mRNA stability and mRNA directly affects various genes, for example, fatty acids synthetase (affects lipid metabolism) and inflammatory response proteins [33].

Polyunsaturated fatty acids regulate the expression of genes in various tissues including liver, heart, adipose tissue and brain [34]. The BDNF (Brain-Derived Neurotrophic Factor) signalling, crucial for synaptic plasticity and neuronal survival, depends on fatty acid intake and composition [20]. Neuronal synaptic plasticity also depends on expression of the genes regulated by calcium signalling. Calcium ions are important second (intra and intercellular) messengers passing cell membranes [35].

The brain is particularly prone to inflammatory and oxidative alterations which may underlie decreases in learning and memory [36]. Neuroinflammation is connected to ROS (reactive oxygen species) production [19], while omega−3 fatty acids reduce neuroinflammation and, thus, regulate expression of genes involved in inflammatory responses (TNF-Tumor Necrosis Factor Alpha and IL−6—Interleukin−6). Neuroinflammation involves mainly microglial cells which modulate synaptic functions through phagocytosis of unnecessary synapses [19]. Oxidative stress is also affected by fatty acid metabolism through the synthesis of free radicals and expression of SOD (Superoxide Dismutase). Decreased levels of polyunsaturated fatty acids result in a decreased rate of incorporation into membranes and a decrease in the activities of delta−6 and delta−9 desaturase enzymes, leading to an increase in free radicals.

In conclusion, the genetic apparatus of neurons responds sensitively to fatty acids from food. They effect the expression level of many genes like synaptic plasticity, cytoskeleton and membrane association, signal transduction and ion channel formation. Still, there is an open question whether they affect brain genome in free form or through effect on membranes as previously explained [31]. However, cognitive processes are very complex and cannot be traced back to a simple accumulation of DHA in neuronal membranes, but to the biophysical properties and structural integrity of all neuronal membranes, including nerve endings, is crucial for brain health [31,37]. It has been observed that a deficiency of essential polyunsaturated fatty acids can cause symptoms like attention deficient/hyperactivity disorder (ADHD) [38].

Brain enzymes, proteins and peptides consist of amino acids derived from dietary proteins. The agents responsible for transmission between neurons are substances eventually formed of essential amino acids supplied by dietary proteins. Consequently, the brain needs a continual supply of amino acids for the synthesis of certain neurotransmitters, notably catecholamines and serotonin [31,37]. The human body does not have a reserve of proteins and they need to be eaten at every meal, especially breakfast [31]. Between 10 and 20% of energy came from proteins in all samples except samples 11 and 12 (29.7 and 27.5%), but by carefully combining them with different samples, it would be very easy to reach ideal values. To make the combinations optimal, all samples should be divided into three groups according to the following fat levels: first group samples with low fat: number 4 and 7; second group samples with the recommended (or very close to the recommended) level of fat: number 1, 3, 11, 12, 13, 14, 15 and 16; and samples with a high level of fat: samples 2, 5, 6, 8, 9, 10. If a student decides to choose one tapa from group three, it should be combined with a tapa from group one and vice versa. This kind of offering would allow flexibility in choices and adjustment to personal preferences and ensure a well-balanced, complete meal.

Numerous micronutrients from the group of vitamins and minerals are associated with cognitive functioning, whereby iron, iodine, magnesium, zinc, selenium, B complex vitamins, vitamin C, vitamin D and vitamin E are the most often mentioned in the literature. They affect memory, attention, recollection, learning, mental fatigue and comprehension through different mechanisms like serving as neurotransmitters (B group vitamins), serving as antioxidants (zinc, selenium, vitamin C, Vitamin E), serving as building elements of nerve endings (vitamin C), involvement in prevention of neurodegenerative diseases (vitamin D), involvement in brain energy production (iron, magnesium) and influence on cerebral development (iodine, irone) [39,40].

Samples 8, 10 and 11 have the highest sodium amount due to cheese and prosciutto. Sodium intake should not exceed recommended value. Especially good sources of selenium were sample numbers 11, 2, 6, 8, 5 and 12 due to sardines and tuna, cheese and nuts. Samples 4 and 11 were excellent source of carotenoids due to red peppers and sweet potatoes. Carotenoids are known as strong antioxidants and it would be good to keep them as a recommendation for combining with other tapas, although only 50.1% (sample 4) and 62.5% of students (sample 11) rated them with the top three ratings on the hedonic scale (Table 4). Sample 16 stood out as the best source of vitamin E (1.1 mg) due to hazelnuts. Besides sample 8, sample 11 provided a particularly good source of vitamin C.

## 4. Conclusions

Although students have a clear attitude about preferences for certain foods, it is possible to expand their eating habits through a wise combination of the ingredients they prefer and those they do not like. This was particularly successful in the case of fish, which students never choose as the option for their morning meal, especially sardines and anchovies. When sardines were combined with cream cheese, olive oil, and spices into a spread served on toast, over 80% of the sensory panel scored it with top three ratings. Similar result was with anchovies when incorporated in tortilla sauce. In that way students can enrich their diet with valuable nutrients like unsaturated fatty acids, selenium and retinol. Red peppers were incorporated in two tapas, which resulted with high level of carotenoids. Dried fruits were also initially scored as undesirable, but when dried dates were mixed into spread together with hazelnuts, cocoa powder, and agave syrup, over 90% of sensory panel scored it with top three ratings. The only key ingredient which led to unacceptable scores by majority of sensory panel was the purple cabbage. To ensure an optimally combined complete meal, we grouped the proposed tapas into three groups according to fat level (high, optimal and low). If a student would choose one tapa from group one, it would be obligatory to choose one from group three and vice versa. This allows certain flexibility, but also ensures an optimally balanced meal. Consuming food with nutritionally rich ingredients that students do not usually consume brings short-term benefits like energy and nutrient supply and long-term benefits in the sense of adoption of a healthy life style.

The following provides future perspectives: research could be expanded in the central university campus, with a larger number of participants because this study was conducted at a faculty with a small number of students, separate from the central university. It could also be expanded in the following other directions: research of antioxidant properties of samples and molecules related to it, sustainability of proposed concept and expansion to new combinations.

## Figures and Tables

**Table 1:** Eating habits of respondents (*n* = 46).

Question	Answer	F	M	χ^2^	*p*-Value	EG	SF	χ^2^	*p*-Value
Are you familiar with tapas?	Yes	12	4	0.4609	0.9274	15	1	7.7652	0.0498
	No	25	5			16	14		
Do you eat breakfast?	Yes	28	8	0.7476	0.8620	28	8	8.1340	0.0433
	No	9	1			3	7		
Do you eat morning snack?	Yes	25	8	1.6169	0.6556	25	8	3.7167	0.2937
	No	12	1			6	7		
Would you like to have tapas for breakfast?	Yes	29	9	2.3657	0.5000	27	11	1.0500	0.7220
	No	8	0			4	4		
Would you like to have tapas for morning snack?	Yes	33	8	0.0006	1.0000	26	15	2.7133	0.4380
	No	4	1			5	0		

F—female, M—male, EG—enogastronomy students, SF—social field students; χ^2^ crit = 3.84, statistical significance *p* < 0.05.

**Table 2:** The acceptability of selected foods as an ingredient of tapas according to categorical hedonic scale of ten degrees (1 extremely unacceptable, 10 extremely acceptable).

Ingredient	All (*n* = 46)	Female (*n* = 37)	Male (*n* = 9)	EG (*n* = 31)	SF (*n* = 15)	*p* *
Raisins	4.35 ± 3.25	4.16 ± 3.20	5.11 ± 3.55	4.06 ± 3.11	4.93 ± 3.58	0.4388 ^a^ 0.4020 ^b^
Honey	6.15 ± 3.05	5.89 ± 3.07	7.22 ± 2.86	5.84 ± 3.26	6.80 ± 2.54	0.2445 ^a^ 0.3214 ^b^
Olive oil	7.46 ± 2.60	7.19 ± 2.54	8.56 ± 2.70	7.68 ± 2.56	7.00 ± 2.70	0.1591 ^a^ 0.4130 ^b^
Egg	8.09 ± 2.55	7.95 ± 2.54	8.60 ± 2.65	7.97 ± 2.75	8.33 ± 2.13	0.4524 ^a^ 0.6531 ^b^
Banana	7.50 ± 3.22	7.68 ± 3.17	6.78 ± 3.19	7.23 ± 3.30	8.07 ± 3.06	0.4588 ^a^ 0.4120 ^b^
Orange	6.78 ± 3.22	6.70 ± 3.26	7.11 ± 3.26	6.19 ± 3.23	8.00 ± 2.95	0.7374 ^a^ 0.0745 ^b^
Apple	7.59 ± 2.96	7.65 ± 3.03	7.33 ± 2.83	7.13 ± 2.99	8.53 ± 2.77	0.7782 ^a^ 0.1334 ^b^
Sardines	4.54 ± 3.13	4.11 ± 2.96	6.33 ± 3.35	4.90 ± 3.13	3.80 ± 3.10	0.0549 ^a^ 0.2674 ^b^
Anchovies	4.50 ± 3.25	4.05 ± 3.14	6.33 ± 3.20	4.65 ± 3.27	4.20 ± 3.30	0.0583 ^a^ 0.6682 ^b^
Poultry	8.00 ± 2.56	7.86 ± 2.63	8.56 ± 2.35	7.74 ± 2.68	8.53 ± 2.29	0.4749 ^a^ 0.3321 ^b^
Olives	5.59 ± 3.59	5.27 ± 3.70	6.89 ± 2.93	5.26 ± 3.57	6.27 ± 3.67	0.2296 ^a^ 0.3782 ^b^
Cucumber	7.87±2.92	7.97 ± 2.95	7.44 ± 2.92	7.65 ± 2.95	8.27 ± 2.91	0.6314 ^a^ 0.5269 ^b^
Tomato	7.63 ± 3.25	7.65 ± 3.23	7.16 ± 3.54	7.23 ± 3.58	8.47 ± 2.33	0.9397 ^a^ 0.2295 ^b^
Peppers	7.96 ± 2.74	7.97 ± 2.78	7.89 ± 2.71	7.81 ± 2.85	8.27 ± 2.58	0.9353 ^a^ 0.5990 ^b^
Broccoli	6.93 ± 2.89	6.70 ± 2.99	7.89 ± 2.32	6.74 ± 2.95	7.33 ± 2.79	0.2736 ^a^ 0.5208 ^b^
Cauliflower	6.50 ± 3.09	6.35 ± 3.13	7.11 ± 3.02	6.52 ± 3.02	6.47 ± 3.34	0.5143 ^a^ 0.9601 ^b^
Eggplant	4.87 ± 3.24	4.54 ± 3.15	6.22 ± 3.42	4.97 ± 2.42	4.67 ± 2.92	0.1645 ^a^ 0.7711 ^b^
Leek	5.89 ± 3.45	5.24 ± 3.45	8.56 ± 1.88	5.87 ± 3.50	5.93 ± 3.47	0.0083 ^a^ 0.9550 ^b^
Sweet potato	6.61 ± 3.21	6.27 ± 3.15	8.00 ± 3.28	6.58 ± 3.41	6.67 ± 2.87	0.1497 ^a^ 0.9333 ^b^
Carrot	7.70 ± 2.70	7.49 ± 2.71	8.56 ± 2.60	7.65 ± 2.78	7.80 ± 2.62	0.2916 ^a^ 0.8576 ^b^
Zucchini	7.17 ± 2.98	7.03 ± 3.11	7.78 ± 2.44	7.16 ± 2.83	7.20 ± 3.38	0.5046 ^a^ 0.9676 ^b^
Nuts	7.37 ± 3.09	7.11 ± 3.14	8.44 ± 2.79	7.55 ± 3.05	7.00 ± 3.25	0.2496 ^a^ 0.5788 ^b^
Chickpeas	6.15 ± 3.33	5.59 ± 3.37	8.44 ± 2.01	6.55 ± 3.16	5.33 ± 3.64	0.0196 ^a^ 0.2509 ^b^
Prosciutto	8.78 ± 2.44	8.76 ± 2.34	8.89 ± 2.98	8.87 ± 2.43	8.60 ± 2.53	0.8861 ^a^ 0.7782 ^b^
Bacon	8.48 ± 2.37	8.49 ± 2.43	8.44 ± 2.24	8.48 ± 2.32	8.47 ± 2.56	0.9626 ^a^ 0.9819 ^b^
Champignons	7.54 ± 2.80	7.30 ± 2.87	8.56 ± 2.40	8.19 ± 2.41	6.20 ± 3.14	0.2311 ^a^ 0.0219 ^b^
Beans	6.30 ± 3.40	5.78 ± 3.43	8.44 ± 2.35	6.71 ± 3.21	5.47 ± 3.74	0.0336 ^a^ 0.2493 ^b^
Lentil	5.50 ± 3.33	5.11 ± 3.43	7.11 ± 2.42	5.55 ± 3.18	5.40 ± 3.74	0.1064 ^a^ 0.8893 ^b^
Cheese	9.07 ± 2.23	9.08 ± 2.15	9.00 ± 2.65	9.19 ± 2.21	8.80 ± 2.31	0.9232 ^a^ 0.5797 ^b^
Milk spread	8.13 ± 2.93	8.24 ± 2.86	7.67 ± 3.35	7.87 ± 3.33	8.67 ± 1.84	0.6025 ^a^ 0.3945 ^b^
Salmon	6.11 ± 3.47	5.78 ± 3.46	7.44 ± 3.40	6.45 ± 3.56	5.40 ± 3.29	0.2015 ^a^ 0.3412 ^b^
Tuna	6.54 ± 3.54	6.38 ± 3.59	7.22 ± 3.49	6.32 ± 3.51	7.00 ± 3.70	0.5279 ^a^ 0.5494 ^b^
Tortilla (wheat)	9.17 ± 1.74	9.05 ± 1.87	9.67 ± 1.00	8.94 ± 2.03	9.67 ± 0.72	0.3499 ^a^ 0.1851 ^b^
White bread	7.67 ± 2.76	7.81 ± 2.72	7.11 ± 3.02	7.26 ± 3.00	8.53 ± 2.0	0.5009 ^a^ 0.1433 ^b^
Whole grain bread	8.89 ± 2.29	9.00 ± 2.24	8.44 ± 2.60	8.81 ± 2.57	9.07 ± 1.62	0.5204 ^a^ 0.7225 ^b^
Whole grain tortilla	8.76 ± 1.86	8.92 ± 1.64	8.11 ± 2.62	8.77 ± 1.98	8.73 ± 1.67	0.2480 ^a^ 0.9454 ^b^

Results are expressed as the mean score ± standard deviation; EG—enogastronomy students, SF—social field students, * t-test for independent variables, ^a^—difference between female and male respondents, ^b^—difference between enogastronomy students and social field students, significance level *p* < 0.05.

**Table 3:** Appearance, smell, taste and texture of samples.

Sensory Test		Sample							
Property	Description	1	2	3	4	5	6	7	8
Colour	Acceptable Non-acceptable	100 ± 0.0 0.00 ± 0.0	90.63 ± 4.4 3.13 ± 4.4	100 ± 0.0 0.00 ± 0.0	93.75 ± 8.8 6.25 ± 8.8	90.63 ± 4.4 9.38 ± 4.4	84.38 ± 4.4 9.38 ± 4.4	96.88 ± 4.4 3.13 ± 4.4	81.25 ± 8.8 18.75 ± 8.8
Smell	Neutral Weak Pronounced	25.84 ± 8.8 18.75 ± 0.0 56.25 ± 8.8	12.50 ± 0.0 40.63 ± 4.4 40.63 ± 4.4	12.50 ± 8.8 28.13 ± 4.4 59.38 ± 13.3	0.00 ± 0.0 21.88 ± 4.4 78.13 ± 4.4	0.00 ± 0.0 9.38 ± 4.4 90.63 ± 4.4	6.25 ± 0.0 12.50 ± 17.7 75.00 ± 17.7	0.00 ± 0.0 3.13 ± 4.4 96.88 ± 4.4	6.25 ± 8.8 40.63 ± 13.3 53.13 ± 4.4
Visual consistency	Smooth Rough	43.75 ± 8.8 56.25 ± 8.8	40.63 ± 4.4 53.13 ± 4.4	50.00 ± 0.0 50.00 ± 0.0	87.50 ± 0.0 12.50 ± 0.0	25.00 ± 0.0 75.00 ± 0.0	71.88 ± 4.4 21.88 ± 4.4	40.63 ± 13.3 59.38 ± 13.3	18.75 ± 8.8 81.25 ± 8.8
Surface	Glossy Matte	71.88 ± 4.4 28.13 ± 4.4	56.25 ± 8.8 37.50 ± 8.8	28.13 ± 13.3 71.88 ± 13.3	68.75 ± 8.8 31.25 ± 8.8	28.13 ± 4.4 71.88 ± 4.4	71.88 ± 4.4 21.88 ± 4.4	53.13 ± 4.4 46.80 ± 4.4	53.13 ± 4.4 46.88 ± 4.4
Bitterness	Not present Weak Pronounced	59.38 ± 4.4 28.13 ± 4.4 12.5 ± 0.0	62.50 ± 8.8 21.88 ± 4.4 9.38 ± 4.4	62.50 ± 0.0 18.75 ± 8.8 18.75 ± 8.8	28.13 ± 4.4 71.88 ± 4.4 0.00 ± 0.0	50.00 ± 8.8 31.25 ± 8.8 18.75 ± 0.0	78.13 ± 4.4 9.38 ± 4.4 6.25 ± 0.0	59.38 ± 13.3 40.63 ± 13.3 0.00 ± 0.0	71.88 ± 4.4 25.00 ± 0.0 3.13 ± 4.4
Mouthfeel	Weak Pronounced	53.13 ± 4.4 46.88 ± 4.4	18.75 ± 8.8 75.00 ± 8.8	37.50 ± 8.8 62.50 ± 8.8	15.63 ± 4.4 84.38 ± 4.4	15.63 ± 4.4 84.38 ± 4.4	3.13 ± 4.4 90.62 ± 4.4	9.38 ± 4.4 90.63 ± 4.4	34.38 ± 13.3 65.62 ± 13.3
Sandiness	Not present Pronounced	21.88 ± 4.4 78.13 ± 4.4	62.50 ± 8.8 31.25 ± 8.8	62.50 ± 0.0 37.50 ± 0.0	75.00 ± 8.8 25.00 ± 8.8	28.13 ± 4.4 71.88 ± 4.4	62.50 ± 8.8 31.25 ± 8.8	43.75 ± 8.8 56.25 ± 8.8	78.13 ± 4.4 21.88 ± 4.4
Mouth consistency	Soft Middle Hard	34.37 ± 4.4 65.63 ± 4.4 0.00 ± 0.00	37.50 ± 8.8 56.25 ± 8.8 0.00 ± 0.0	65.63 ± 4.4 25.00 ± 8.8 9.38 ± 4.4	43.75 ± 0.0 46.88 ± 4.4 9.38 ± 4.4	21.88 ± 4.4 53.13 ± 4.4 25.00 ± 0.0	43.75 ± 8.8 37.50 ± 0.0 12.50 ± 8.8	25.00 ± 8.8 68.75 ± 8.8 6.28 ± 0.0	15.63 ± 4.4 18.75 ± 8.8 65.63 ± 13.3
Saltiness	Weak Pronounced	71.88 ± 4.4 28.13 ± 4.4	53.13 ± 4.4 40.63 ± 4.4	84.38 ± 4.4 15.63 ± 4.4	81.25 ± 0.0 18.75 ± 0.0	68.75 ± 8.8 31.25 ± 8.8	37.50 ± 8.8 56.25 ± 8.8	96.88 ± 4.4 3.13 ± 4.4	43.75 ± 8.8 56.25 ± 8.8
Acidity	Weak Pronounced	68.75 ± 0.0 31.25 ± 0.0	65.63 ± 4.4 28.13 ± 4.4	93.75 ± 8.8 6.25 ± 8.8	81.25 ± 0.0 18.75 ± 0.0	18.75 ± 8.8 81.25 ± 8.8	78.13 ± 4.4 15.63 ± 4.4	100.00 ± 0.0 0.00 ± 0.0	100.00 ± 0.0 0.00 ± 0.0
Astringency	Not present Present	81.25 ± 0.0 18.75 ± 0.0	81.25 ± 0.0 12.50 ± 0.0	78.13 ± 4.4 21.88 ± 4.4	56.25 ± 8.8 43.75 ± 8.8	53.13 ± 4.4 46.88 ± 4.4	56.25 ± 8.8 37.50 ± 8.8	56.25 ± 8.8 43.75 ± 8.8	78.13 ± 4.4 21.88 ± 4.4
Hot	Weak Pronounced	96.88 ± 4.4 3.13 ± 4.4	59.38 ± 13.3 34.38 ± 13.3	90.63 ± 4.4 9.38 ± 4.4	12.50 ± 8.8 87.50 ± 8.8	25.00 ± 0.0 75.00 ± 0.0	84.38 ± 4.4 9.38 ± 4.4	96.88 ± 4.4 3.13 ± 4.4	96.88 ± 4.4 3.13 ± 4.4
Spicy	Weak Pronounced	71.88 ± 4.4 28.12 ± 4.4	3.13 ± 4.4 90.63 ± 4.4	53.13 ± 4.4 46.88 ± 4.4	9.38 ± 4.4 90.63 ± 4.4	12.50 ± 8.8 87.50 ± 8.8	46.88 ± 4.4 46.88 ± 4.4	78.13 ± 4.4 21.88 ± 4.4	71.88 ± 4.4 28.13 ± 4.4
Sweetness	Weak Pronounced	100 ± 0.0 0.00 ± 0.0	93.75 ± 0.0 0.00 ± 0.0	53.13 ± 4.4 46.88 ± 4.4	31.25 ± 8.8 68.75 ± 8.8	84.38 ± 4.4 15.63 ± 4.4	93.75 ± 0.0 0.00 ± 0.0	21.88 ± 4.4 78.13 ± 4.4	56.25 ± 8.8 43.75 ± 8.8
Aftertaste	Weak Pronounced Changes with time	46.88 ± 4.4 28.13 ± 4.4 25.00 ± 0.0	37.50 ± 17.7 37.50 ± 8.8 18.75 ± 8.8	12.50 ± 0.0 53.13 ± 4.4 34.38 ± 4.4	3.13 ± 4.4 78.13 ± 13.3 18.75 ± 8.8	9.38 ± 4.4 62.50 ± 8.8 28.13 ± 13.3	12.50 ± 8.84 46.88 ± 4.42 34.38 ± 4.42	3.13 ± 4.42 62.50 ± 0.00 34.38 ± 4.42	40.63 ± 13.26 28.13 ± 4.42 31.25 ± 8.84
Viscosity	Light Dense	36.38 ± 4.4 65.63 ± 4.4	12.50 ± 8.8 81.25 ± 8.8	25.00 ± 8.8 75.00 ± 8.8	28.13 ± 4.4 71.88 ± 4.4	15.63 ± 4.4 84.38 ± 4.4	21.88 ± 4.42 71.88 ± 4.42	3.13 ± 4.42 96.88 ± 4.42	18.75 ± 8.84 81.25 ± 8.84
**Sensory Test**		**Sample**							
**Property**	**Description**	**9**	**10**	**11**	**12**	**13**	**14**	**15**	**16**
Colour	Acceptable Non-acceptable	81.25 ± 8.8 18.75 ± 8.8	100.00 ± 0.0 0.00 ± 0.0	90.63 ± 4.4 9.13 ± 4.4	93.75 ± 0.0 0.00 ± 0.0	93.75 ± 8.8 6.25 ± 8.8	100.00 ± 0.0 0.00 ± 0.0	96.88 ± 4.4 3.13 ± 4.4	100.00 ± 0.0 0.00 ± 0.0
Smell	Neutral Weak Pronounced	25.00 ± 0.0 25.00 ± 0.0 50.00 ± 0.0	25.00 ± 0.0 21.88 ± 4.4 53.13 ± 4.4	3.13 ± 4.4 18.75 ± 0.0 71.88 ± 4.4	43.75 ± 8.8 40.63 ± 4.4 9.38 ± 4.4	12.50 ± 0.0 18.75 ± 8.8 68.75 ± 8.8	87.50 ± 8.8 12.50 ± 8.8 0.00 ± 0.0	9.38 ± 13.2 12.50 ± 8.8 78.12 ± 22.1	18.75 ± 8.8 18.75 ± 8.8 62.50 ± 17.7
Visual consistency	Smooth Rough	53.13 ± 4.4 46.88 ± 4.4	78.13 ± 4.4 21.88 ± 4.4	28.13 ± 4.4 65.63 ± 4.4	53.13 ± 4.4 40.63 ± 4.4	46.88 ± 4.4 53.13 ± 4.4	46.88 ± 4.4 53.13 ± 4.4	56.25 ± 8.8 43.75 ± 8.8	84.38 ± 13.3 15.63 ± 13.3
Surface	Glossy Matte	43.75 ± 8.8 56.25 ± 8.8	56.25 ± 8.8 43.75 ± 8.8	71.88 ± 4.4 21.88 ± 4.4	9.38 ± 4.4 84.38 ± 4.4	21.88 ± 4.4 78.13 ± 4.4	9.38 ± 4.4 90.63 ± 4.4	78.13 ± 4.4 21.88 ± 4.4	81.25 ± 17.7 18.75 ± 17.7
Bitterness	Not present Weak Pronounced	18.75 ± 0.0 31.25 ± 8.8 50.00 ± 8.8	65.63 ± 22.1 31.25 ± 26.5 3.13 ± 4.4	25.00 ± 0.0 53.13 ± 4.4 15.63 ± 4.4	81.25 ± 8.8 12.50 ± 8.8 0.00 ± 0.0	90.63 ± 4.4 9.38 ± 4.4 0.00 ± 0.0	84.38 ± 13.3 15.63 ± 13.3 0.00 ± 0.0	78.13 ± 4.4 21.88 ± 4.4 0.00 ± 0.	87.50 ± 8.8 12.50 ± 8.8 0.00 ± 0.0
Mouthfeel	Weak Pronounced	50.00 ± 0.0 50.00 ± 0.0	40.63 ± 13.3 59.38 ± 13.3	31.25 ± 0.0 62.50 ± 0.0	28.13 ± 4.4 65.63 ± 4.4	71.88 ± 4.4 28.13 ± 4.4	96.88 ± 4.4 3.13 ± 4.4	50.00 ± 0.0 50.00 ± 0.0	31.25 ± 8.8 68.75 ± 8.8
Sandiness	Not present Pronounced	65.63 ± 13.2 34.38 ± 13.2	93.75 ± 8.8 6.25 ± 8.8	56.25 ± 8.8 37.50 ± 8.8	68.75 ± 8.8 25.00 ± 8.8	84.38 ± 13.3 15.63 ± 13.3	65.63 ± 13.3 34.38 ± 13.3	65.63 ± 22.1 34.38 ± 22.1	53.13 ± 4.4 46.88 ± 4.4
Mouth consistency	Soft Middle Hard	56.25 ± 8.8 40.63 ± 13.3 3.13 ± 4.4	53.13 ± 4.4 46.88 ± 4.4 0.00 ± 0.0	28.13 ± 4.4 46.88 ± 4.4 8.75 ± 0.0	43.75 ± 8.8 37.50 ± 0.0 12.50 ± 8.8	68.75 ± 8.8 31.25 ± 8.8 0.00 ± 0.0	56.25 ± 8.8 28.13 ± 4.4 15.63 ± 13.3	53.13 ± 4.4 46.88 ± 4.4 0.00 ± 0.0	81.25 ± 17.7 18.75 ± 17.7 0.00 ± 0.0
Saltiness	Weak Pronounced	68.75 ± 8.8 31.25 ± 8.8	37.50 ± 17.7 62.50 ± 17.7	43.75 ± 0.0 50.00 ± 0.0	31.25 ± 8.8 62.50 ± 8.8	96.88 ± 4.4 3.13 ± 4.4	84.38 ± 13.3 15.63 ± 13.3	100.0 ± 0.0 0.00 ± 0.0	100.0 ± 0.0 0.00 ± 0.0
Acidity	Weak Pronounced	96.87 ± 4.4 3.13 ± 4.4	56.25 ± 8.8 43.75 ± 8.8	71.88 ± 4.4 21.88 ± 4.4	90.63 ± 4.4 3.13 ± 4.4	100.0 ± 0.0 0.00 ± 0.	93.75 ± 0.0 6.25 ± 0.00	53.13 ± 4.4 46.88 ± 4.4	100.0 ± 0.0 0.00 ± 0.0
Astringency	Not present Present	78.13 ± 4.4 21.88 ± 4.4	96.88 ± 4.4 3.13 ± 4.4	81.25 ± 8.8 12.50 ± 8.8	84.38 ± 4.4 9.38 ± 4.4	81.25 ± 8.8 18.75 ± 8.8	90.63 ± 4.4 9.38 ± 4.4	93.75 ± 8.8 6.25 ± 8.8	96.88 ± 4.4 3.13 ± 4.4
Hot	Weak Pronounced	59.38 ± 13.3 40.63 ± 13.3	68.75 ± 8.8 31.25 ± 8.8	81.25 ± 8.8 12.50 ± 8.8	68.75 ± 8.8 25.00 ± 8.8	96.88 ± 4.4 3.13 ± 4.4	96.88 ± 4.4 3.13 ± 4.4	96.88 ± 4.4 3.13 ± 4.4	90.63 ± 13.3 9.38 ± 13.3
Spicy	Weak Pronounced	53.13 ± 4.4 46.88 ± 4.4	28.13 ± 4.4 71.88 ± 4.4	53.13 ± 4.4 40.63 ± 4.4	46.88 ± 4.4 46.88 ± 4.4	53.13 ± 4.4 46.88 ± 4.4	100.0 ± 00.0 0.00 ± 0.0	78.13 ± 4.4 21.88 ± 4.4	65.63 ± 13.3 34.38 ± 13.3
Sweetness	Weak Pronounced	100 ± 0.0 0.00 ± 0.0	90.63 ± 4.4 9.31 ± 4.4	81.25 ± 8.8 12.50 ± 8.8	90.63 ± 4.4 3.13 ± 4.4	68.75 ± 8.8 31.25 ± 8.8	96.88 ± 4.4 3.13 ± 4.4	46.88 ± 4.4 53.13 ± 4.4	28.13 ± 4.4 71.88 ± 4.4
Aftertaste	Weak Pronounced Changes with time	37.50 ± 8.8 46.88 ± 4.4 15.63 ± 13.3	34.38 ± 4.4 50.00 ± 17.6 15.63 ± 13.2	28.13 ± 13.3 43.75 ± 8.8 21.88 ± 4.4	37.50 ± 0.0 43.75 ± 8.8 12.50 ± 8.8	50.00 ± 0.0 28.13 ± 4.4 21.88 ± 4.4	93.75 ± 8.8 3.13 ± 4.4 3.13 ± 4.4	46.88 ± 4.4 46.88 ± 4.4 6.25 ± 8.8	21.88 ± 13.3 65.63 ± 13.3 12.50 ± 0.0
Viscosity	Light Dense	18.75 ± 8.8 81.25 ± 8.8	31.25 ± 8.8 68.75 ± 8.8	28.13 ± 4.4 65.63 ± 4.4	18.75 ± 0.0 75.00 ± 0.0	18.75 ± 8.8 81.25 ± 8.8	12.50 ± 8.8 87.50 ± 8.8	71.88 ± 4.4 28.13 ± 4.4	21.88 ± 13.3 78.13 ± 13.3

Results represent the percentage of respondents who rated a particular attribute, expressed as the mean of two repetitions ± standard deviation.

**Table 4:** Nine-point categorical hedonic scale (1—especially highly undesirable to 9—especially highly desirable).

	Sample															
	1	2	3	4	5	6	7	8	9	10	11	12	13	14	15	16
9	3.1 ^ab^	15.6 ^bcd^	9.4 ^abc^	0.0 ^a^	9.4 ^abc^	18.8 ^cd^	31.3 ^de^	25.0 ^d^	9.4 ^abc^	46.9 ^e^	9.4 ^ab^	68.8 ^e^	3.1 ^ab^	0.0 ^a^	18.8 ^cd^	46.9 ^e^
8	37.5 ^bc^	40.6 ^bc^	31.3 ^abc^	31.3 ^abc^	15.6 ^ab^	40.6 ^bc^	25.0 ^abc^	43.8 ^b^	18.8 ^abc^	40.6 ^bc^	37.5 ^bc^	6.3 ^a^	34.4 ^bc^	6.3 ^a^	31.3 ^ab^	37.5 ^bc^
7	25.0 ^bc^	25.0 ^bc^	25.0 ^bc^	18.8 ^ab^	21.9 ^b^	18.8 ^ab^	21.9 ^b^	21.9 ^b^	31.3 ^b^	6.3 ^a^	15.6 ^ab^	15.6 ^ab^	25.0 ^bc^	46.9 ^c^	18.8 ^ab^	9.4 ^a^
6	9.4 ^ab^	12.5 ^ab^	6.3 ^ab^	6.3 ^ab^	9.4 ^ab^	3.1 ^a^	18.8 ^ab^	9.4 ^ab^	15.6 ^ab^	3.1 ^a^	12.5 ^ab^	0.0 ^a^	21.9 ^b^	18.8 ^ab^	15.6 ^ab^	0.0 ^a^
5	12.5 ^ab^	0.0 ^a^	12.5 ^ab^	3.1 ^a^	18.8 ^b^	9.4 ^ab^	3.1 ^a^	0.0 ^a^	12.5 ^ab^	3.1 ^a^	6.3 ^ab^	3.1 ^a^	9.4 ^ab^	28.1 ^b^	0.0 ^a^	0.0 ^a^
4	9.4 ^ab^	0.0 ^a^	9.4 ^ab^	12.5 ^b^	6.3 ^ab^	3.1 ^ab^	0.0 ^a^	0.0 ^a^	12.5 ^b^	0.0 ^a^	15.6 ^b^	0.0 ^a^	9.4 ^ab^	0.0 ^a^	0.0 ^a^	0.0 ^a^
3	0.0 ^a^	0.0 ^a^	0.0 ^a^	18.8 ^a^	9.4 ^a^	0.0 ^a^	0.0 ^a^	0.0 ^a^	0.0 ^a^	0.0 ^a^	0.0 ^a^	0.0 ^a^	0.0 ^a^	0.0 ^a^	6.3 ^a^	3.1 ^a^
2	0.0 ^a^	0.0 ^a^	6.3 ^a^	9.4 ^a^	9.4 ^a^	0.0 ^a^	0.0 ^a^	0.0 ^a^	0.0 ^a^	0.0 ^a^	0.0 ^a^	0.0 ^a^	0.0 ^a^	0.0 ^a^	3.1 ^a^	3.1 ^a^
1	0.0 ^a^	0.0 ^a^	0.0 ^a^	0.0 ^a^	0.0 ^a^	0.0 ^a^	0.0 ^a^	0.0 ^a^	0.0 ^a^	0.0 ^a^	0.0 ^a^	0.0 ^a^	0.0 ^a^	0.0 ^a^	6.3 ^a^	0.0 ^a^

Results represent the percentage of respondents for each grade, expressed as the mean of two repetitions. Means followed by the same letter in the rows are not statistically different at 5% probability.

**Table 5:** Energy value and macronutrients in samples.

Sample	Energy (kcal)	Carbohydrates (g)/Ratio in Energy Value of the Meal (%)	Proteins (g)/Ratio in Energy Value of the Meal (%)	Fats (g)/Ratio in Energy Value of the Meal (%)
1.	85.6	13.1/57.3	4.4/20.5	2.1/22.1
2.	112.9	12.7/42	4.6/16.3	5.2/41.5
3.	105.2	13.9/49.5	3.8/14.4	4.2/35.9
4.	75.7	14.7/71.8	3.0/15.6	1.1/12.9
5.	113.8	14.2/46.7	4.3/15.1	4.9/38.6
6.	119.7	12.7/39.8	4.5/15.1	6.0/45.2
7.	104.7	18.4/67.1	4.2/16.3	1.9/16.6
8.	210.6	13.2/23.5	12.1/23	12.6/53.8
9.	137.1	12.9/35.2	6.8/19.8	6.9/45.1
10.	140.7	11.9/31.8	7.5/21.4	7.3/46.8
11.	130.3	17.4/49.9	9.7/29.7	3.0/20.6
12.	222.0	20.5/36.2	14.6/27.5	8.6/36.4
13.	79.6	10.7/47.3	3.2/15.1	3.5/37.2
14.	41.0	6.1/50.6	2.2/19.5	1.5/29.9
15.	67.5	9.1/47.6	2.6/14.5	3.0/37.7
16.	98.5	13.9/50.8	3.0/11.7	4.3/37.7

Results are expressed per one serving, Recommended ratio in energy value from carbohydrates is 45–65%, from proteins 10–20% and from fats 20–35%.

**Table 6:** Selected micronutrients in samples.

	Fiber (g)	SF (g)	MUF (g)	PUF (g)	Cholesterol (mg)	Na (mg)	Ca (mg)	Zn (mg)	Se (µg)	I (µg)	Retinol (µg)	Carotene (µg)	Vit E (mg)	Vit C (mg)
RDA/*UL	25	*24.4	36.6	19.5	*300	1500	1000	8.0	46	150	800	-	15	75
1.	1.6	0.75	0.57	0.49	3.8	190.1	59.2	0.63	3.4	3.1	8.3	17.7	0.11	0.0052
2.	1.5	2.8	1.6	0.41	11.2	195.8	42.1	0.58	7.3	0.86	35.1	24.6	0.23	0.29
3.	2.1	2.3	1.0	0.46	8.5	144.1	35.7	0.61	0.58	1.9	38.3	58.2	0.19	2.3
4.	1.8	0.41	0.19	0.27	0.0	138.6	34.4	0.53	2.9	0.52	0.0	467.8	0.21	10.6
5.	2.0	0.91	2.6	0.93	0.0	167.1	40.7	0.92	5.0	1.1	0.0	24.3	0.20	5.0
6.	1.5	3.0	2.0	0.58	12.3	202.3	71.9	0.66	6.1	1.5	34.2	24.6	0.23	0.29
7.	2.2	0.41	0.54	0.61	-	140.4	38.6	0.64	2.9	0.018	-	4	0.31	-
8.	1.6	6.6	2.9	1.9	29.4	372.9	198.7	1.6	5.4	5.0	97.3	84.2	0.48	80
9.	1.8	7.3	5.8	1.4	12.5	182.8	41.4	0.71	4.5	1.3	8.2	10.9	0.41	0.065
10.	1.3	3.6	1.7	0.58	17.1	264.7	139.7	0.95	1.6	4.2	54.7	58.9	0.44	1.2
11.	2.6	0.75	0.93	0.82	14.3	295.8	64.0	0.95	17.0	25.3	2.8	401.4	0.88	50.9
12.	0.27	2.9	2.5	1.1	34.2	190.2	64.6	0.6	5.1	6.8	23.5	35.9	0.96	4.5
13.	2.0	1.6	0.77	0.8	29.4	156.8	39.7	0.33	2.5	4.0	9.9	3.5	0.23	0.71
14.	0.48	0.46	0.53	0.32	23.4	62.7	18.2	0.27	1.9	4.7	12.2	1.2	0.13	Tr
15.	0.91	0.63	0.77	1.3	23.4	66.3	22.6	0.35	1.9	4.9	12.2	4.2	0.26	0.72
16.	1.2	0.95	2.4	0.56	23.4	64.1	30.5	0.41	2.2	7.0	12.2	2.4	1.1	Tr

RDA—recommended daily allowance, UL—upper level, SF—saturated fats, MUF—monounsaturated fats, PUF—polyunsaturated fats.

## Data Availability

The data presented in this study are available on request from the corresponding author. The data are not publicly available due to privacy restrictions.

## Supplementary Materials

The following supporting information can be downloaded at: https://www.mdpi.com/article/10.3390/foods13213432/s1, Table S1: Ingredients and preparation of tapas.
