# Supplementary material for: Development of Nutrient Rich Morning Meals for Students by Concept of Tapas
Source: Foods. 2024 Oct 28;13(21):3432. doi: 10.3390/foods13213432 (PMC11545402; doi:10.3390/foods13213432)
Supplement: Supplementary file 1 [file foods-13-03432-s001.zip › foods-3272382-supplementary.pdf]

Table S1. Ingredients and preparation of tapas

Tapas	Ingredients for 10 servings	Preparation
1. Cheese spread with toast	- Bread, integral, toasted 250 g<br>- Cottage cheese, plain 130 g<br>- Yogurt from partially skimmed milk (3.2 % milk fat) 65 g<br>- Sesame seeds 3.9 g<br>- Flax seeds 3.9 g<br>- Salt 0.85 g<br>- Chili powder 0.86 g	Sesame seeds were briefly fried on a heated pan until golden, and crushed together with flax seeds in a kitchen multipurpose processor.<br>Crushed seeds were mixed with yogurt, cheese and spices, and the spread was served on toasted bread.
2. Tuna spread with toast	- Bread, integral, toasted 250 g<br>- Tuna, canned in brine, drained 61 g<br>- Cream cheese, classic 87 g<br>- Onion 43 g<br>- Lemon juice, freshly squeezed 3 g<br>- Olive oil 3 g<br>- Salt 0.87 g<br>- Pepper, black 0.87 g<br>- Parsley, dry 0.22 g<br>- Shallot 2.2 g	The onion was cut into small pieces and mixed with the other ingredients. The mixture was mixed with a hand stick mixer to a creamy texture. The spread was served on toasted bread.
3. Pea butter with toast	- Bread, integral, toasted 250 g<br>- Butter, unsalted 40 g<br>- Peas, boiled in unsalted water, drained 133 g<br>- Onion 36 g<br>- Salt 0.67 g<br>- Pepper, black 0.67 g<br>- Lemon zest 0.67 g	The peas were boiled in salted water, drained, transferred to ice water to cool down and drained again. The onion was sautéed in butter. All ingredients were mixed in a kitchen blender until a thick creamy texture. The spread was served on toasted bread.
4. Sweet potato and pepper spread with toast	- Bread, integral, toasted 250 g<br>- Sweet potato, boiled in salted water 100 g<br>- Coconut oil 3 g<br>- Garlic, cut 1 g<br>- Chili powder 0.54 g<br>- Salt 0.4 g<br>- Pepper, black 0.4 g<br>- Basil, fresh 0.1 g<br>- Pepper, red, boiled in unsalted water 100 g	The sweet potato was peeled, cut into cubes, boiled in water for 20 minutes and drained.<br>Red peppers were baked in the oven at 200 °C for 30 minutes and peeled from the skin. The garlic was fried in coconut oil. All ingredients were mixed in a kitchen blender until a thick creamy texture. The spread was served on toasted bread.
5. Purple cabbage spread with toast	- Bread, integral, toasted 250 g<br>- Cashew nut 72 g<br>- Cabbage, purple 100 g<br>- Olive oil 6 g<br>- Garlic, cut 6 g<br>- Lemon juice, freshly squeezed 4 g<br>- Salt 1.2 g<br>- Almond milk 8 g	All ingredients were mixed in a kitchen blender until a thick creamy texture. The spread was served on toasted bread.
6. Sardine spread with toast	- Bread, integral, toasted 250 g	All ingredients were mixed in a kitchen blender until a thick creamy

	- Sardines, preserved in olive oil, drained 61 g<br>- Cream cheese, classic 87 g<br>- Onion 43 g<br>- Lemon juice, freshly squeezed 3 g<br>- Olive oil 3 g<br>- Salt 0.87 g<br>- Pepper, black 0.87 g<br>- Parsley, dry 0.22 g<br>- Shallot 2.2 g	texture. The spread was served on toasted bread.
7. Chocolate hummus with toast	- Bread, integral, toasted 250 g<br>- Agave syrup 52 g<br>- Cocoa powder 8 g<br>- Peanut butter, smooth 16 g<br>- Almond milk 12 g<br>- Chickpeas, boiled in unsalted water 98 g<br>- Vanilla extract 1.6 g<br>- Salt 0.4 g	All ingredients were mixed in a kitchen blender until a thick creamy texture. The spread was served on toasted bread.
8. Cheese bruschetta	- Bread, integral, toasted 250 g<br>- Cheese, Camembert 100 g<br>- Cheese, Edam100 g<br>- Cheese, Brie, peeled 100 g<br>- Cheese, Mozzarella, fresh 100 g<br>- Grapes, green 50 g<br>- A walnut 25 g<br>- Pine nut 5 g	Each of the four types of cheese was put on thin slices of bread and baked in the oven until the cheese melted. Pine nuts, walnuts and grapes were added on the baked cheese toast before serving.
9. Chicken mousse with toast	- Bread, integral, toasted 250 g<br>- Chicken, breast, skinless 109 g<br>- Pistachios, fried and salted 43 g<br>- Cheese, Mascarpone 22 g<br>- Onion 22 g<br>- Vinegar, balsamic 0.87 g<br>- Olive oil 22 g<br>- Salt 0.87 g<br>- Pepper, black 0.87 g	The onions were fried in oil until golden and then stewed together with chicken. When the chicken was sufficiently stewed, it was seasoned with salt and pepper. The cooled chicken was blended together with the other ingredients, and mixed into a smooth mass in a kitchen blender. The spread was served on toasted bread.
10. Tortilla with prosciutto	- Tortilla, wheat, soft 163 g<br>- Prosciutto 56 g<br>- Tomato28 g<br>- Parsley, leaf, fresh, chopped 2.2 g<br>- Cheese, Cheddar 110 g<br>- Yogurt, Greek, sheep's milk (6 % milk fat) 167 g<br>- Oregano, dry, chopped 0.56 g<br>- Lettuce salad 28 g<br>- Kidney beans, red, preserved in water, heated, drained 139 g<br>- Salt 1.1 g	The tortilla was coated with yogurt seasoned with oregano and salt, and other ingredients were placed on it. The tortilla was then rolled and cut into smaller pieces.
11. Peppers and sardines on toast	- Capers, canned 22 g<br>- Olive oil 2.9 g	Halves of peppers were placed on the baking sheet, salted and peppered.

	- Sardines (boneless) 280 g<br>- Salt 1.7 g<br>- Pepper, black 1.1 g<br>- Basil, dry 0.56 g<br>- Bread, wholemeal 350 g<br>- Pepper, red, boiled in unsalted water 556 g<br>- Zucchini, cut 278 g<br>- cottage cheese, plain 139 g	The capers were finely chopped and sprinkled on the peppers. One sardine was placed on each half of the pepper. Everything was sprinkled with olive oil and baked at 200 °C for about 20 minutes. Zucchini was cut into slices, seasoned with salt, pepper, dry basil, drizzled with olive oil, and baked on a grill pan. Toasted bread was spread with fresh cheese, baked zucchini, and peppers with sardines were put on top of it.
12. Tortilla with Caesar sauce	- Chicken, breast, skinless, grilled 250 g<br>- Cheese, Parmesan, fresh 50 g<br>- Anchovies preserved in oil, drained 12 g<br>- Capers, canned 10 g<br>- Pancetta, fried 21 g<br>- Mayonnaise, reduced amount of fat 83 g<br>- Sour cream, 12 % milk fat 42 g<br>- Lemon juice, freshly squeezed 2.1 g<br>- Cauliflower, flowers, boiled in unsalted water 125 g<br>- Tomato 21 g<br>- Lettuce salad 21 g<br>- tortilla 333 g	Mayonnaise, sour cream, anchovies, capers, fried pancetta, parmesan and lemon juice were mixed together with a stick mixer into creamy sauce. Cauliflower was cut into smaller pieces and boiled briefly, chicken meat was cut into strips and fried on a pan. The tortillas were first coated with sauce, then the salad, cauliflower, chicken and tomatoes were added, tortillas were rolled up and cut into smaller pieces.
13. Buckwheat and apple muffins	- Apple, peeled 167 g<br>- Egg, whole 78 g<br>- Whole spelled flour 83 g<br>- Yogurt from partially skimmed milk (3.2 % milk fat) 150 g<br>- Peanut butter, smooth 8.4 g<br>- Cinnamon, ground 5.8 g<br>- Baking powder 5.7 g<br>- Salt 1.7 g<br>- Coconut flour 17 g<br>- Flax seeds 17 g	First, the yolks were separated from the whites. Then, egg yolks were mixed with sugar to a foamy texture. Peanut butter, yogurt and dry ingredients (flour, salt, cinnamon and baking powder) were added, and mixed again until a homogeneous mixture.<br>The egg whites were beaten until stiff and slowly added to the mixture with the yolks. At the end, apples cut into small cubes were mixed in. Before baking (in muffin molds), the muffins were sprinkled with ground flax seeds and coconut. Muffins were baked in the oven at 180 °C for 40 minutes.
14. Oatmeal and cheese pancakes	- Oatmeal 67 g<br>- Fresh cheese, plain 67 g<br>- Egg, whole 63 g<br>- Baking powder 3.1 g	All ingredients were mixed in a blender until a thick homogeneous mixture. The mixture was scooped out onto a pan heated on medium heat, and baked until golden on both sides.

15. Oatmeal and cheese pancakes with apple spread	- Oatmeal 67 g<br>- Fresh cheese, plain 67 g<br>- Egg, whole 63 g<br>- Baking powder 3.1 g<br>- Apple, peeled 167 g<br>- Walnuts 22 g<br>- Maple syrup, golden 11 g<br>- Cinnamon, ground 1.6 g<br>- Lemon juice, freshly squeezed 1.1 g	Oatmeal, cheese, eggs and baking powder were mixed in a blender until a thick homogeneous mixture. The mixture was scooped out onto a pan heated on medium heat, and baked until golden on both sides.<br>For apple spread all ingredients were mixed with kitchen blender until creamy texture.
16. Oatmeal and cheese pancakes with cocoa and hazelnut spread	- Oatmeal 67 g<br>- Fresh cheese, plain 67 g<br>- Egg, whole 63 g<br>- Baking powder 3.1 g<br>- Dates, dry 100 g<br>- Hazelnut 36 g<br>- Cocoa powder 4 g<br>- Agave syrup 20 g<br>- Reduced fat coconut milk 40 g	Oatmeal, cheese, eggs and baking powder were mixed in a blender until a thick homogeneous mixture. The mixture was scooped out onto a pan heated on medium heat, and baked until golden on both sides.<br>For cocoa and hazelnut spread all ingredients were mixed with kitchen blender until creamy texture.
